# Modelling the cost effectiveness of non-alcoholic fatty liver disease risk stratification strategies in the community setting

**DOI:** 10.1371/journal.pone.0251741

**Published:** 2021-05-21

**Authors:** Stephen E. Congly, Abdel Aziz Shaheen, Mark G. Swain

**Affiliations:** 1 Division of Gastroenterology and Hepatology, Department of Medicine, Cumming School of Medicine, University of Calgary, Calgary Alberta, Canada; 2 O’Brien Institute of Public Health, University of Calgary, Calgary Alberta, Canada; 3 Department of Community Health Sciences, University of Calgary, Calgary Alberta, Canada; Policlinico Universitario Campus Bio-Medico, ITALY

## Abstract

**Background:**

Non-alcoholic fatty liver disease (NAFLD) is highly prevalent worldwide. Identifying high-risk patients is critical to best utilize limited health care resources. We established a community-based care pathway using 2D ultrasound shear wave elastography (SWE) to identify high risk patients with NAFLD. Our objective was to assess the cost-effectiveness of various non-invasive strategies to correctly identify high-risk patients.

**Methods:**

A decision-analytic model was created using a payer’s perspective for a hypothetical patient with NAFLD. FIB-4 [≥1.3], NAFLD fibrosis score (NFS) [≥-1.455], SWE [≥8 kPa], transient elastography (TE) [≥8 kPa], and sequential strategies with FIB-4 or NFS followed by either SWE or TE were compared to identify patients with either significant (≥F2) or advanced fibrosis (≥F3). Model inputs were obtained from local data and published literature. The cost/correct diagnosis of advanced NAFLD was obtained and univariate sensitivity analysis was performed.

**Results:**

For ≥F2 fibrosis, FIB-4/SWE cost $148.75/correct diagnosis while SWE cost $276.42/correct diagnosis, identifying 84% of patients correctly. For ≥F3 fibrosis, using FIB-4/SWE correctly identified 92% of diagnoses and dominated all other strategies. The ranking of strategies was unchanged when stratified by normal or abnormal ALT. For ≥F3 fibrosis, the cost/correct diagnosis was less in the normal ALT group.

**Conclusions:**

SWE based strategies were the most cost effective for diagnosing ≥F2 fibrosis. For ≥F3 fibrosis, FIB-4 followed by SWE was the most effective and least costly strategy. Further evaluation of the timing of repeating non-invasive strategies are required to enhance the cost-effective management of NAFLD.

## Introduction

Non-alcoholic fatty liver disease (NAFLD) affects approximately 25% of the population [1], with a spectrum of disease ranging from simple steatosis, non-alcoholic steatohepatitis, advanced fibrosis and cirrhosis. Making management challenging is that the gold standard for diagnosis of non-alcoholic steatohepatitis and staging fibrosis is liver biopsy, which is not a feasible primary risk stratification strategy. NAFLD is associated with increased morbidity and mortality [2, 3] and is projected to be the leading indication for liver transplant in the near future [4]. Given the significant burden of disease, the health-care system is at risk of becoming overwhelmed [5]. Notably, only a small proportion of patients are at increased risk of progression to advanced fibrosis [6], and given limited health care resources, these patients are key targets for identification and early, more aggressive liver-focused intervention. Multiple NAFLD risk stratification pathways recommend using non-invasive strategies [7, 8]. Using sensitive non-invasive strategies to identify patients with advanced fibrosis is appealing for evaluating the population at risk, given the resources and risks associated with biopsy; however, any test strategy should be cost-effective. A number of strategies have been proposed including using lab-based tests such as the fibrosis-4 (FIB-4) and the NAFLD fibrosis score (NFS) [9–11], as well as elastography [12–14].

Given the significant number of patients with NAFLD in our referral catchment area (~1.4 million people), the Calgary Liver Unit, in partnership with local Primary Care Networks developed a clinical care pathway for managing patients at risk for, or with documented non-alcoholic fatty liver disease. Within this pathway, patients at risk for NAFLD are referred by their primary care physician to have an abdominal ultrasound with shear wave elastography. Patients at low risk for significant liver fibrosis stay in the primary care medical home, while patients thought to be “at risk” for significant liver fibrosis are referred to hepatology for formal assessment. Our goal with this study was to assess the cost-effectiveness of an ultrasound-based elastography strategy developed to streamline only at-risk patients with NAFLD to specialist care and compare this strategy to other potential risk stratification strategies for identifying patients at risk of clinically significant fibrosis.

## Materials and methods

### Calgary NAFLD pathway

In 2016, a clinical care pathway was created to improve management of patients with NAFLD in the Calgary Zone as a partnership involving the University of Calgary Liver Unit, the largest Calgary radiology service provider and the Calgary Primary Care Networks. Within the pathway, primary care physicians were encouraged to refer their patients who were at risk for having NAFLD [having risk factors including obesity, diabetes, hyperlipidemia, or metabolic syndrome (if not captured by the preceding risk factors); or the presence of elevated liver enzymes or steatosis on ultrasound] to undergo an abdominal ultrasound with shear wave elastography to assess liver stiffness as an estimate of the severity of fibrosis. The presence of other chronic liver diseases including significant alcohol consumption (>1 standard drink/day for women, >2 per day for men), immune-mediated liver disorders or viral hepatitis were excluded from the pathway [15]. Patients with liver stiffness values ≥8 kPa or indeterminate scans were recommended to be referred to hepatology, while those at low risk for significant fibrosis based on shear wave elastography result (values <8 kPa) were recommended to stay in their primary care medical home, cared for using management recommendations provided within the NAFLD pathway. Repeat shear wave elastography was recommended every three years for low-risk patients to monitor for changes in liver stiffness, with a score ≥8 kPa triggering a subsequent referral to hepatology at that time. A shear wave elastography cut-off of ≥8 kPa was chosen as the negative predictive value of advanced fibrosis for values <8 kPa exceeds 96% [11, 16]. A schematic of the Calgary NAFLD pathway [15] is included (S1 Fig).

All patients undergoing shear wave elastography as part of the NAFLD pathway were included in a database which included information on patient characteristics [age, sex, body mass index (BMI)], comorbidities (including presence of metabolic syndrome, diabetes, tobacco use, dyslipidemia, hypertension) and laboratory data including alanine aminotransferase (ALT), aspartate aminotransferase (AST), alkaline phosphatase (ALP), albumin, and gamma glutamyl transpeptidase (GGT). FIB-4 values and NFS were calculated when relevant laboratory data was available. For patients formally seen in the NAFLD hepatology clinic, transient elastography [FibroScan^™^ (EchoSens, Paris)] was performed as part of standard of care. Patients seen by hepatology and felt to be at high risk for advanced liver fibrosis were offered liver biopsy for assessment of disease activity and staging at the clinician’s discretion.

### Patient characteristics

Data from 1,958 patients evaluated within the Calgary NAFLD pathway were used to inform this model, with 30% of the patients having normal liver enzymes (n = 577). A total of 5856 patients have been enrolled in the pathway from January 2018-December 2019 [15]. Full details regarding patient characteristics in the Calgary non-alcoholic fatty liver disease pathway included in the analysis are found in Table 1. A total of 167 patients were eligible for review in the NAFLD clinic, 113 patients were formally evaluated and 32 underwent a liver biopsy. Liver biopsies showed 1 patient with F0 fibrosis, 4 F1 fibrosis, 4 F2 fibrosis, 12 F3 fibrosis and 11 F4 fibrosis.

**Table 1:** Demographic details of NAFLD risk stratification group.

Characteristic	NAFLD patients without elevated liver enzymes N = 577 (29.7%)	NAFLD patients with elevated liver enzymes N = 1,367 (70.3%)	P Value
Age, years	57 (48–66)	54 (43–62)	<0.001
Female sex	55.6% (321)	53.1% (726)	0.308
BMI (kg/height in m^2^)	31.8 (27.8–36.5)	32.0 (28.2–36.6)	0.495
Baseline investigations			
ALT, U/L	19 (15–23)	49 (36–70)	<0.001
AST, U/L	19 (16–23)	35 (26–47)	<0.001
Albumin, g/L	38 (36–40)	40 (37–42)	<0.001
Alkaline phosphatase, U/L	74 (62–91)	78 (65–95)	0.001
GGT, U/L	26 (18–45)	56 (34–104)	<0.001
INR	1.0 (1.0–1.1)	1.0 (1.0–1.0)	0.356
Platelets, 10E9/L	255 (213–300)	247 (206–289)	0.005
Triglycerides, mmol/L	1.68 (1.19–2.40)	1.87 (1.31–2.71)	<0.001
Cholesterol, mmol/L	4.57 (3.85–5.23)	4.83 (4.09–5.57)	<0.001
HDL, mmol/L	1.20 (0.99–1.40)	1.14 (0.94–1.37)	0.006
LDL, mmol/L	2.53 (1.83–3.09)	2.66 (1.98–3.37)	<0.001
Creatinine, mmol/L	74 (60–86)	74 (60–87)	0.84
A1C, %	5.7 (5.4–6.2)	5.7 (5.5–6.3)	0.178
Diabetes	26.1% (164)	28.5% (410)	0.251
Glucose intolerance	33.4% (210)	32.9% (472)	0.81
Hypertension	41.7% (262)	38.9% (559)	0.239
FIB-4	0.97 (0.69–1.33)	1.01 (0.69–1.55)	0.215
FIB-4 cut-off 1.30	26.60%	33.60%	0.016
FIB-4 cut-off 1.45	21.10%	28.00%	0.014
NAFLD Fibrosis Score	-0.71 (-1.70, 0.33)	-1.24 (-2.14, -0.08)	<0.001
NFS cut-off -1.455	69.20%	54.70%	<0.001
NFS cut-off 0.675	18.80%	13.30%	0.042
SWE	4.3 (3.6–5.2)	4.5 (3.8–5.6)	<0.001
SWE ≥7 kPa	4.50%	5.80%	0.257
SWE ≥8 kPa	3.30%	3.50%	0.809
Inconclusive	6.40%	4.50%	0.085

Distribution is expressed as median (interquartile range) or percentage (number).

BMI, body mass index; ALT, alanine aminotransferase; AST, aspartate aminotransferase; ALP, alkaline phosphatase; GGT, gamma glutamyl transpeptidase; INR, international normalized ratio; HDL, high density lipoprotein; LDL, low density lipoprotein; HbA1c, hemoglobin A1c; FIB-4, fibrosis-4 variable index; NFS, NAFLD fibrosis score; SWE, shear wave elastography.

This study was approved by the Conjoint Health Research Ethics Board at the University of Calgary. Requirement of informed consents by participants was waived by the ethics review committee. All patients’ data were deidentified and anonymized after linkage.

### Cost-effectiveness analysis

Decision-tree models were created to evaluate several strategies to identify patients in the community with NAFLD with increased risk for either significant (defined as having ≥F2 fibrosis) or advanced fibrosis (defined as having ≥F3 fibrosis) in the community using TreeAge Pro 2020 (TreeAge Software, Williamstown MA). Risk stratification strategies for evaluation of at risk patients were a) NAFLD fibrosis score (increased risk >-1.455), b) FIB-4 (increased risk >1.30) c) shear wave elastography (increased risk >8 kPa), d) transient elastography (increased risk >8 kPa), e) FIB-4 followed by shear wave elastography (if FIB-4 ≥1.30), f) NAFLD fibrosis score followed by shear wave elastography (if NAFLD fibrosis score >-1.455), g) FIB-4 followed by transient elastography (if FIB-4 >1.30) and h) NAFLD fibrosis score followed by transient elastography (if NAFLD fibrosis score >-1.455). Thresholds for at risk values were based on previously validated and recommended values in the literature [7, 11, 17, 18].

In these models, it was assumed that patients who were identified to be potentially at increased risk of significant fibrosis by these non-invasive strategies were subsequently referred for in-person hepatology evaluation with transient elastography performed, if not already done, and then all referred patients were presumed to undergo a confirmatory liver biopsy (assumed to be the gold standard for defining significant fibrosis). The primary endpoint was the cost per correct diagnosis of fibrosis stage which is being used as a surrogate marker of cost-effectiveness. A false positive was considered as being an unnecessary referral to hepatology for a patient without significant/advanced fibrosis, while a false negative was considered as a patient with significant/advanced fibrosis that was not referred to hepatology. The baseline analyses were used to identify patients at increased risk for significant (*>*F2) and advanced fibrosis (>F3). Subsequently, patients were stratified as to the presence or absence of abnormal liver enzymes at any time within the previous two years [ALT >30 in men, ALT >25 in women] [19], and scenario analyses regarding the cost-effectiveness of identifying patients with significant or advanced fibrosis stratified by liver enzyme levels were performed. Univariate analysis of both base-case analyses was performed.

Data regarding test characteristics come from the literature. Costs of physician assessment, lab tests and diagnostic imaging come from Alberta Health and Wellness and the prevalence rates of testing are from our program (Table 2). A public payer’s perspective was taken; discounting was not performed as this was a one-time screening strategy. Costs are reported as 2019 Canadian dollars. Where required, costs were adjusted to 2019 Canadian dollars through use of the Bank of Canada inflation calculator [20].

**Table 2:** Base case scenario analysis variables.

Variable	Base Case	Low Range	High Range	Reference
**Population Characteristics**				
Proportion of patients with negative FIB-4 (<1.30)	0.684	0.550	0.750	Local data
Proportion of patients with negative NFS (<-1.45)	0.414	0.300	0.500	Local data
Proportion of patients with negative SWE (<8 kPa)	0.956	0.850	0.980	Local data
Proportion of patients with positive SWE given positive FIB-4	0.086	0	0.300	Local data
Proportion of patients with positive SWE given positive NFS	0.061	0	0.300	Local data
Proportion of patients with negative TE (<8 kPa)	0.866	0.800	0.900	[7]
Proportion of patients with positive TE given positive FIB-4	0.330	0.250	0.350	[7, 14]
Proportion of patients with positive TE given positive NFS	0.359	0.300	0.400	[14]
**F2 Fibrosis Test Characteristics**				
Negative predictive value of FIB-4	0.606	0.405	0.742	[11]
Negative predictive value NFS	0.736	0.611	0.860	[11]
Negative predictive value of SWE	0.848	0.825	0.870	[11]
Negative predictive value of TE	0.788	0.720	0.840	[11]
Positive predictive value of FIB-4	0.733	0.662	0.778	[11]
Positive predictive value of NFS	0.817	0.766	0.867	[11]
Positive predictive value of SWE	0.939	0.878	1	[11]
Positive predictive value of TE	0.655	0.540	0.830	[11]
**F3 Fibrosis Test Characteristics**				
Negative predictive value of FIB-4	0.927	0.880	0.980	[11]
Negative predictive value NFS	0.918	0.813	0.981	[11]
Negative predictive value of SWE	0.934	0.926	0.942	[11]
Negative predictive value of TE	0.887	0.840	0.930	[11]
Positive predictive value of FIB-4	0.403	0.240	0.506	[11]
Positive predictive value of NFS	0.504	0.240	1	[11]
Positive predictive value of SWE	0.882	0.833	0.931	[11]
Positive predictive value of TE	0.587	0.450	0.710	[11]
**Costs**				
Cost of clinic visit [$]	220.00	180.00	250.00	Alberta Health and Wellness
Cost of FIB-4 test [$]	17.00	10.00	25.00	Alberta Health and Wellness
Cost of transient elastography [$]	125.00	50.00	200.00	[21]
Cost of NAFLD fibrosis score [$]	22.00	15.00	30.00	Alberta Health and Wellness
Cost of shear wave elastography [$]	198.90	150.00	250.00	Alberta Health and Wellness
Cost of a liver biopsy	540.83	400.00	700.00	[22]

All dollar values are 2019 Canadian dollars.

FIB-4, Fibrosis-4; NFS, NAFLD fibrosis score; SWE, shear wave elastography; TE, transient elastography.

We used the Consolidated Health Economic Evaluation Reporting Standards (CHEERS) checklist when writing our manuscript [23] (S1 Appendix).

## Results

### Cost-effectiveness

#### F2 fibrosis

**Fig 1:**
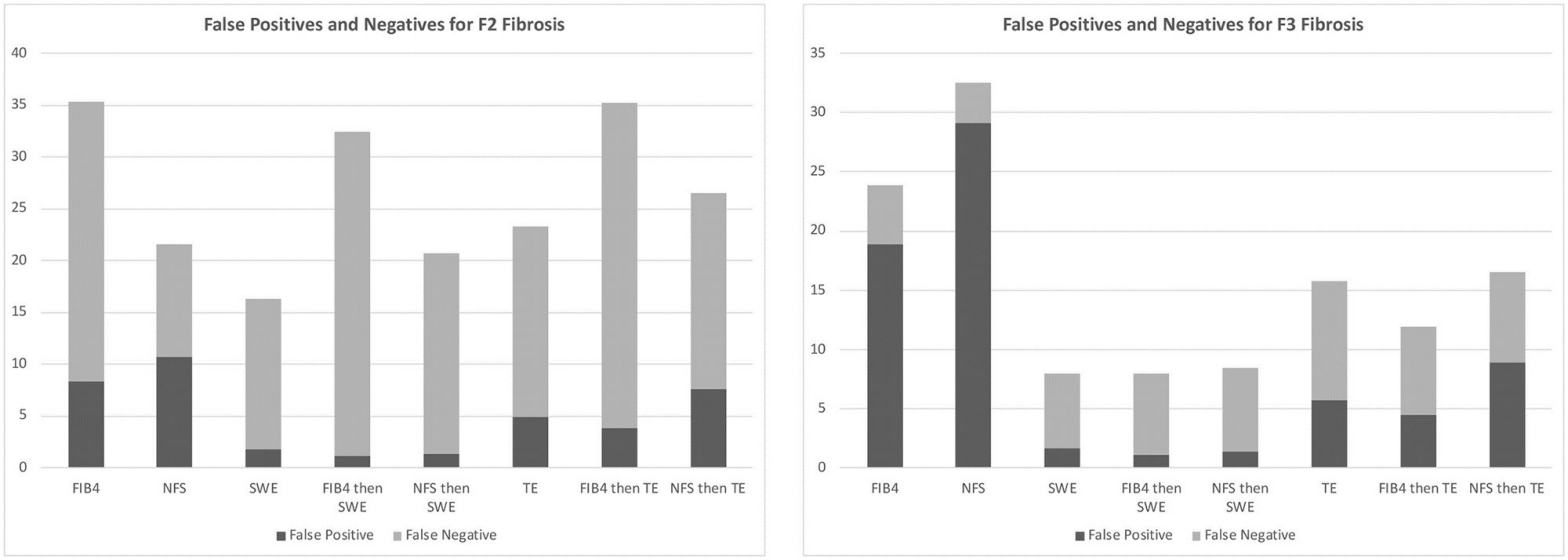
Classification of incorrect diagnoses by risk stratification strategy. Stratification of incorrect diagnoses based on risk strategies. False positive is considered as being an unnecessary referral for a patient without significant/advanced fibrosis while a false negative was a patient with significant/advanced fibrosis that was not referred to a hepatologist.

In the base case scenario for identifying >F2 fibrosis in all patients (Table 3), a FIB-4/shear wave elastography strategy cost $100.53 and obtained the correct diagnosis 67.6% of the time. Shear wave elastography alone correctly identified 84% of cases at an incremental cost of $137.35 versus FIB-4/shear wave elastography. Use of FIB-4 or the NAFLD fibrosis score alone, as well as any transient elastography based methods, were less effective and more costly than other strategies and so were considered to be dominated. With the use of FIB-4 based one-time strategies, there was a high miss rate of over 25% (Fig 1).

**Table 3:** Cost-effectiveness of finding F2 fibrosis in all patients.

Strategy	Cost [$]	Incremental Cost [$]	Effectiveness [Correct Diagnosis]	Incremental Effectiveness [Correct Diagnosis]	Incremental Cost Effectiveness Ratio (ICER) [$/Correct Diagnosis]
FIB-4/SWE	100.53	-	0.6758	-	-
FIB-4/TE	135.84	31.91	0.6479	-0.0279	**DOMINATED**
NFS/SWE	170.22	66.29	0.7929	0.1171	566.35
TE	226.95	123.03	0.7679	0.0921	**DOMINATED**
SWE	237.88	133.95	0.8372	0.1614	2557.68
NFS/TE	255.31	151.38	0.7350	0.0592	**DOMINATED**
FIB-4	296.92	193.00	0.6461	-0.0297	**DOMINATED**
NFS	541.10	437.17	0.7835	0.1077	**DOMINATED**
Biopsy all	885.83	781.90	1	0.3242	2411.81

All dollar values are 2019 Canadian dollars.

FIB-4, Fibrosis-4; NFS, NAFLD fibrosis score; SWE, shear wave elastography; TE, transient elastography.

#### F3 fibrosis

FIB-4/shear wave elastography was the preferred strategy for risk stratifying all referred patients for F3 fibrosis with a cost of $100.93 for 92% correct diagnoses. All other strategies were dominated except for the gold standard of liver biopsy (Table 4). The FIB4/shear wave elastography sequential strategy would miss 7% of patients with advanced fibrosis and see an additional 2% of patients without significant fibrosis (Fig 1).

**Table 4:** Cost-effectiveness of finding F3 fibrosis in all patients.

Strategy	Cost [$]	Incremental Cost [$]	Effectiveness [Correct Diagnosis]	Incremental Effectiveness [Correct Diagnosis]	Incremental Cost Effectiveness Ratio (ICER) [$/Correct Diagnosis]
FIB-4/SWE	103.93	-	0.9204	-	-
FIB-4/TE	135.84	31.91	0.8819	-0.0385	**DOMINATED**
NFS/SWE	170.22	66.29	0.9157	-0.0046	**DOMINATED**
TE	226.95	123.03	0.8452	-0.0751	**DOMINATED**
SWE	237.88	133.95	0.9197	-0.0007	**DOMINATED**
NFS/TE	255.31	151.38	0.8343	-0.0861	**DOMINATED**
FIB-4	296.92	193.00	0.7614	-0.1590	**DOMINATED**
NFS	541.10	437.17	0.6754	-0.2450	**DOMINATED**
Biopsy all	885.83	781.90	1	0.0796	9818.81

All dollar values are 2019 Canadian dollars.

FIB-4, Fibrosis-4; NFS, NAFLD fibrosis score; SWE, shear wave elastography; TE, transient elastography.

#### Normal vs. abnormal liver enzymes

When patients referred to the program were stratified based on normal or abnormal liver enzymes (ALT >30 in men, and >25 in women considered abnormal), for identification of >F2 fibrosis the ranking of risk stratification strategies was identical to the base-case analysis, although the incremental cost effectiveness ratio (ICER) between strategies was higher in the normal ALT scenario (S1 and S2 Tables). For >F3 fibrosis, FIB-4/shear wave elastography remained the dominant strategy for non-invasive risk stratification for both the normal and abnormal ALT patient populations. Notably, FIB-4/shear wave elastography had a lower cost/correct diagnosis in patients with a normal ALT, and higher cost/correct diagnosis in those patients with an abnormal ALT, versus all patients (S3 and S4 Tables). In the abnormal ALT scenario, the shear wave elastography strategy became extendedly dominated (the incremental cost-effectiveness ratio was higher than a more effective strategy). The rates of missed diagnosis were similar compared to the base-case scenarios for both F2 and F3 (S2 and S3 Figs).

#### Sensitivity analysis

Univariate sensitivity analysis for the F2 model showed the variables impacting the model were the rate of patients with both a positive NAFLD fibrosis score and shear wave elastography, the negative predictive value of the NAFLD fibrosis score, and the negative predictive value of transient elastography. For F3 fibrosis, the model was sensitive to the prevalence of patients with a negative FIB-4, as well as the negative predictive value of the FIB-4, NAFLD fibrosis score and shear wave elastography. Neither model was impacted by cost. Full details of the sensitivity analysis are found in Table 5.

**Table 5:** Univariate sensitivity analysis.

F2 fibrosis			
**Variable**	**Base Case**	**Threshold**	**Impact**
Proportion of patients with positive SWE given positive NFS	0.061	>0.204	NFS/SWE becomes dominated
		>0.162	TE no longer dominated
NPV NFS	0.736	>0.852	SWE becomes dominated
		<0.671	TE no longer dominated
NPV TE	0.655	>0.827	TE no longer dominated
F3 fibrosis			
Proportion of patients with positive SWE given positive FIB-4	0.086	>0.117	SWE no longer dominated
		>0.141	NFS/SWE no longer dominated
		>0.200	FIB-4/TE no longer dominated
Proportion with negative FIB-4	0.684	<0.669	SWE no longer dominated
Proportion with negative SWE	0.956	>0.963	SWE no longer dominated
Proportion of patients with positive SWE given positive NFS	0.061	<0.052	NFS/SWE no longer dominated
NPV FIB-4	0.927	<0.922	SWE no longer dominated
		<0.918	NFS/SWE no longer dominated
NPV NFS	0.918	>0.924	NFS/SWE no longer dominated
NPV SWE	0.934	>0.935	SWE no longer dominated
PPV NFS	0.504	>0.918	NFS no longer dominated

SWE = Shear wave elastography; NFS = NAFLD fibrosis score, NPV = negative predictive value, PPV = positive predictive value.

### Budget impact analysis

For a budget impact analysis, the complete total of 5856 patients enrolled into the non-alcoholic fatty liver disease pathway for risk stratification from January 2018-December 2019 was used. Assuming that all patients were able to be seen in consultation by a hepatologist and underwent a confirmatory liver biopsy, the budget impact would be $5.2 million over the two years of the program studied. Through the one-time use of the non-alcoholic fatty liver disease pathway with elastography to identify patients with advanced fibrosis, the savings exceed $3.8 million compared to seeing and biopsying all patients. Further savings would be found with use of the most cost-effective FIB-4/shear wave elastography approach where an additional $784,000 could be saved by adding in FIB-4 for risk stratification.

## Discussion

In our cost-effectiveness model for risk stratifying non-alcoholic fatty liver disease patients in the community, we found that all evaluated strategies were more accurate at identifying F3 fibrosis compared to F2 fibrosis. To identify patients with F2 fibrosis, use of shear wave elastography based strategies were preferred with the highest accuracy seen with shear wave elastography alone, given the poorer test characteristics of FIB-4, the NAFLD fibrosis score and transient elastography for F2 fibrosis. In F3 fibrosis, the dominant strategy was FIB-4 followed by shear wave elastography; shear wave elastography strategies correctly identified over 90% of advanced fibrosis.

Current expert recommendations [12, 24–26] for evaluation of NAFLD in the primary care setting suggest initial risk stratification with FIB-4 or NAFLD fibrosis score, then referral of all at-risk patients for further evaluation and elastography. The test characteristics of FIB-4 and the NAFLD fibrosis score require further confirmatory testing if they are positive; there are a significant proportion of indeterminate tests that require further evaluation. Other two-step approaches that have been studied include performing FIB-4 followed by transient elastography [7, 27], NAFLD fibrosis score followed by transient elastography [28], FIB-4 followed by enhanced liver fibrosis (ELF^™^) serum testing [8] and transient elastography followed by FibroMeter^®^ [27]. We found that a two-step strategy with shear wave elastography was reasonable for identifying patients with advanced fibrosis and was modelled to have an absolute reduction in the number of specialist referrals by 27%, compared to using FIB-4 as the sole risk stratification tool, and 54% using the NAFLD fibrosis score as the sole risk stratification tool. For identifying patients with an increased likelihood of significant fibrosis, shear wave elastography was the dominant strategy. Notably, our model demonstrated that shear wave elastography was superior to transient elastography in all base case and scenario analyses.

There have been several recent studies looking at sequential combinations of liver fibrosis assessment, given the challenges associated with only using a single testing modality. In a British hepatology clinic, patients were initially risk stratified with FIB-4 and patients with high-risk scores (>3.25) were recommended to be referred to hepatology, with low-risk patients (<1.30) staying in primary care. Patients with indeterminate results were further assessed with the serum-based ELF^™^ test. With this pathway, there was an absolute reduction in low yield referrals to hepatology by 22% [8]. Similarly, in a Canadian series modelling initial risk stratification with FIB-4, with values >1.30 having transient elastography as a confirmatory test, only 15% were deemed high risk based on FIB-4 and 4% required hepatology review [7]. The Calgary NAFLD pathway found that approximately 7% of patients assessed were at high risk.

Several economic models have been created on the cost-effectiveness of strategies for risk stratifying patients with non-alcoholic fatty liver disease for advanced fibrosis—laboratory testing followed by transient elastography for confirmation [28–30], transient elastography with magnetic resonance elastography [13, 31] and transient elastography alone [32, 33]; all of which identify the use of any non-invasive strategy to identify advanced fibrosis as being cost-effective compared to biopsy. Similarly, we found the use of non-invasive methods of risk stratification were also cost-effective at identifying high risk patients and reducing low value referrals to speciality care and are among the first to analyze the cost-effectiveness of shear-wave elastography for NAFLD. With our comparison of the most commonly used non-invasive tests for determining those patients with non-alcoholic fatty liver disease at risk for advanced fibrosis and adding shear wave elastography which may potentially be more broadly accessible than transient elastography at the community level, this analysis of non-invasive strategies is a strength of our study. Our study complements the cost-effectiveness of a two-step approach for NAFLD risk stratification and highlights the challenges of relying on only one test and is unique in that it compares shear wave elastography and transient elastography. Further, this study is the first to analyze risk stratification strategies for patients with NAFLD with normal liver enzymes, which is an important population to consider given that 19% of patients with NAFLD have normal liver tests [34].

In our analysis, we have chosen an endpoint of cost per correct diagnosis as a pragmatic way to economically evaluate each screening strategy [35]. Standard practice for patients in the community with NAFLD thought to be at increased risk of advanced fibrosis would be referral to a specialist for evaluation and confirmation of this diagnosis. The risk with any non-invasive evaluation strategy is that patients falsely deemed to be high risk of advanced fibrosis have low value care and testing while patients falsely felt to be low risk of advanced fibrosis may be missed. As such, identifying the cost per correct diagnosis is a relevant endpoint to best utilize health care resources.

Through our budget impact analysis, we demonstrate the significant cost and burden of NAFLD in our centre which is similar to other jurisdictions. Targeting high risk patients is critical to maximize health care resource utilization, given the huge potential economic implications of NAFLD [36]. In Canada, NAFLD is estimated to affect 25% of the adult population, equivalent to 7.6 million people. Through the use of the FIB-4/shear wave elastography strategy, there would be approximately $5.9 billion in savings versus specialist assessment of all patients.

Our study has significant strengths and provides information to fill the health economic knowledge gap regarding risk stratification of NAFLD patients. Our model is powered by a large North American real-world primary care cohort reflecting the heterogeneity of NAFLD referrals. Previous studies have been based out of Europe and Asia, and to date, there has not been any modelling from a North American primary care perspective. In our series, we included patients with both normal and abnormal liver enzymes who were at increased risk for NAFLD. Use of elevated ALT alone to identify patients with NAFLD may miss a significant number of patients, as almost 20% of patients with NASH and 25% with NAFLD have a normal ALT [34]. We were able to model the impact of risk stratifying patients with advanced fibrosis in a wide spectrum of NAFLD patients, including the important subset with normal ALT, and show that the ranking of strategies was unchanged, although the cost of risk stratifying strategies is lower in patients with normal liver enzymes. Our study is generalizable given our evaluation of non-proprietary tests, ultrasound-based shear wave elastography as well as transient elastography in the form of FibroScan^™^. The majority of studies to date have modeled the use of transient elastography for risk stratification, typically using FibroScan^™^ with few using transient elastography. However, FibroScan^™^ is most commonly located in speciality clinics and subsequently access may be more limited, especially in smaller centres. In contrast, shear wave elastography is easily integrated into performing an abdominal ultrasound, which is more widely accessible in the community.

In our study, we analyzed the cost effectiveness of identifying both significant and advanced fibrosis with one-time testing. Ideally, early intervention in patients with significant fibrosis would lead to regression and improved outcomes. However, at this point, there are currently no approved therapies for NAFLD and so treatment options beyond lifestyle are limited. Therefore, given the current test characteristics and population burden of NAFLD, we currently suggest targeting patients with advanced fibrosis (>F3) as the priority for specialist assessment. In the future, with improved tests, risk stratification of at-risk patients could be expanded to those with F2 fibrosis. Given the limited number of patients with cirrhosis identified, we do not have the data to formally analyze the best testing strategy for this cohort but would suggest that given F3 fibrosis is the precursor to cirrhosis, using the F3 strategy would be appropriate; further studies are required in this subpopulation.

Given imperfections of the non-invasive diagnosis of fibrosis stage, no strategy is completely perfect, leading to either excess specialist consultation or patients that are being missed. As the sensitivity of the testing strategy increases (less low value consultation), more patients may be missed and vice versa. Obtaining the optimal screening test is an important consideration for a risk-stratifying program. Given the relative low risk of fibrosis progression for most patients with NAFLD (about one stage every seven years) [6], repeat testing will identify the majority of cases, similar to colon cancer screening and the use of fecal immunochemistry testing. In the long term, repeated risk stratification will be required to ensure patients are not missed, and to follow fibrosis progression. Further data will inform the optimal interval for repeat testing; given the slow progression of NAFLD, likely every three years is a reasonable interval. Our results provide some guidance as to the appropriate test to use in the community setting.

Our study does have some limitations. We were not able to include other proprietary NAFLD fibrosis blood tests, such as the ELF^™^ score or FibroSURE^®^, as these were not available to patients in our centre. We modelled the risk stratification strategy itself as a one-time strategy as patients in our program have not yet returned for repeat risk stratification, which would help model fibrosis progression over time. Our model is based on meta-analysis of the characteristics of testing modalities which typically are derived from speciality care settings, and so may not fully apply to the community setting. A limited number of patients were biopsied in the cohort of patients seen in clinic and so it is possible that the performance characteristics of the non-invasive strategies in our population do not match the results found in the literature, although we think this is unlikely. Moreover, our model is based on Canadian costing; the cost-effectiveness of strategies may vary based on the cost of testing, although our model would likely show similar results in the United States given the similar cost of abdominal ultrasound with elastography and transient elastography [37, 38].

## Conclusions

Overall, implementing a wide risk-stratification program for NAFLD patients to identify significant fibrosis with non-invasive testing was cost effective. Using a two-step risk stratification strategy with shear wave elastography provided the highest rate of accuracy and was the most cost effective, although importantly, no testing strategy is perfect. Recurrent testing will be essential as a longitudinal method of following these patients, although further research into the most appropriate testing interval is still required.

## Supporting information

S1 Table Cost-effectiveness of finding F2 fibrosis in patients with normal ALT. (DOCX)

S2 Table Cost-effectiveness of finding F2 fibrosis in patients with abnormal ALT. (DOCX)

S3 Table Cost-effectiveness of finding F3 fibrosis in patients with normal ALT. (DOCX)

S4 Table Cost-effectiveness of finding F3 fibrosis in patients with abnormal ALT. (DOCX)

S1 Fig Calgary NAFLD care pathway. Schematic of the Calgary NAFLD care pathway.
(TIF)

S2 Fig Classification of incorrect diagnoses by risk stratification strategy in normal ALT subgroup. Stratification of incorrect diagnoses based on risk strategies. False positive is considered as being an unnecessary referral for a patient without significant/advanced fibrosis while a false negative was a patient with significant/advanced fibrosis that was not referred to a hepatologist.
(TIF)

S3 Fig Classification of incorrect diagnoses by risk stratification strategy in abnormal ALT subgroup. Stratification of incorrect diagnoses based on risk strategies. False positive is considered as being an unnecessary referral for a patient without significant/advanced fibrosis while a false negative was a patient with significant/advanced fibrosis that was not referred to a hepatologist.
(TIF)

S1 Appendix CHEERS checklist. (DOCX)
