# Supplementary material for: Modelling the cost effectiveness of non-alcoholic fatty liver disease risk stratification strategies in the community setting
Source: PLoS One. 2021 May 21;16(5):e0251741. doi: 10.1371/journal.pone.0251741 (PMC8139490; doi:10.1371/journal.pone.0251741)
Supplement: S1 Table — (DOCX) [file pone.0251741.s001.docx]

S1 Table: Cost-Effectiveness of Finding F2 Fibrosis in Patients with Normal ALT

| Strategy | Cost [$] | Incremental Cost  [$] | Effectiveness  [Correct Diagnosis] | Incremental Effectiveness  [Correct Diagnosis] | Incremental Cost Effectiveness Ratio (ICER)  [$/Correct Diagnosis] |
| --- | --- | --- | --- | --- | --- |
| FIB-4/SWE | 85.22 | - | 0.6661 | - | - |
| FIB-4/TE | 117.04 | 31.81 | 0.6413 | -0.0249 | **DOMINATED** |
| NFS/SWE | 185.38 | 100.16 | 0.8064 | 0.1402 | 714.20 |
| TE | 226.95 | 41.57 | 0.7679 | -0.0384 | **DOMINATED** |
| SWE | 237.88 | 52.49 | 0.8372 | 0.0308 | 1702.62 |
| FIB-4 | 252.63 | 14.75 | 0.6398 | -0.1974 | **DOMINATED** |
| NFS/TE | 297.51 | 59.63 | 0.7348 | -0.1024 | **DOMINATED** |
| NFS | 634.99 | 397.12 | 0.7921 | -0.0451 | **DOMINATED** |
| Biopsy all | 885.83 | 647.95 | 1 | 0.1628 | 3980.07 |

All dollar values are 2019 Canadian dollars.

FIB-4, Fibrosis-4; NFS, NAFLD fibrosis score; SWE, shear wave elastography; TE, transient elastography

y.
