# Supplementary material for: Modelling the cost effectiveness of non-alcoholic fatty liver disease risk stratification strategies in the community setting
Source: PLoS One. 2021 May 21;16(5):e0251741. doi: 10.1371/journal.pone.0251741 (PMC8139490; doi:10.1371/journal.pone.0251741)
Supplement: S2 Table — (DOCX) [file pone.0251741.s002.docx]

S2 Table: Cost-Effectiveness of Finding F2 Fibrosis in Patients with Abnormal ALT

| Strategy | Cost [$] | Incremental Cost  [$] | Effectiveness  [Correct Diagnosis] | Incremental Effectiveness  [Correct Diagnosis] | Incremental Cost Effectiveness Ratio (ICER)  [$/Correct Diagnosis] |
| --- | --- | --- | --- | --- | --- |
| FIB-4/SWE | 111.21 | - | 0.6797 | - | - |
| FIB-4/TE | 143.36 | 32.15 | 0.6506 | -0.0292 | **DOMINATED** |
| NFS/SWE | 164.72 | 53.50 | 0.7879 | 0.1081 | 494.76 |
| TE | 226.95 | 115.74 | 0.7679 | 0.0882 | **DOMINATED** |
| SWE | 237.88 | 126.66 | 0.8372 | 0.1575 | 804.34 |
| NFS/TE | 239.78 | 128.57 | 0.7351 | 0.0553 | **DOMINATED** |
| FIB-4 | 314.64 | 203.43 | 0.6487 | -0.0311 | **DOMINATED** |
| NFS | 506.55 | 395.34 | 0.7803 | 0.1006 | **DOMINATED** |
| Biopsy all | 885.83 | 774.62 | 1 | 0.3203 | 2418.60 |

All dollar values are 2019 Canadian dollars.
FIB-4, Fibrosis-4; NFS, NAFLD fibrosis score; SWE, shear wave elastography; TE, transient elastography
