# Supplementary material for: Modelling the cost effectiveness of non-alcoholic fatty liver disease risk stratification strategies in the community setting
Source: PLoS One. 2021 May 21;16(5):e0251741. doi: 10.1371/journal.pone.0251741 (PMC8139490; doi:10.1371/journal.pone.0251741)
Supplement: S3 Table — (DOCX) [file pone.0251741.s003.docx]

S3 Table: Cost-Effectiveness of Finding F3 Fibrosis in Patients with Normal ALT

| Strategy | Cost [$] | Incremental Cost  [$] | Effectiveness  [Correct Diagnosis] | Incremental Effectiveness  [Correct Diagnosis] | Incremental Cost Effectiveness Ratio (ICER)  [$/Correct Diagnosis] |
| --- | --- | --- | --- | --- | --- |
| FIB-4/SWE | 85.22 | - | 0.9232 | - | - |
| FIB-4/TE | 117.04 | 31.81 | 0.8890 | -0.0342 | **DOMINATED** |
| NFS/SWE | 185.38 | 100.16 | 0.9196 | -0.0036 | **DOMINATED** |
| TE | 226.95 | 141.73 | 0.8452 | -0.0780 | **DOMINATED** |
| SWE | 237.88 | 152.65 | 0.9197 | -0.0036 | **DOMINATED** |
| FIB-4 | 252.63 | 167.41 | 0.7876 | -0.1356 | **DOMINATED** |
| NFS/TE | 297.51 | 212.29 | 0.8191 | -0.1041 | **DOMINATED** |
| NFS | 634.99 | 549.77 | 0.6315 | -0.2917 | **DOMINATED** |
| Biopsy all | 885.83 | 800.61 | 1 | 0.0768 | 10,429.24 |

All dollar values are 2019 Canadian dollars.
FIB-4, Fibrosis-4; NFS, NAFLD fibrosis score; SWE, shear wave elastography; TE, transient elastography
