# Supplementary figures and images for: Modelling the cost effectiveness of non-alcoholic fatty liver disease risk stratification strategies in the community setting
Source: PLoS One. 2021 May 21;16(5):e0251741. doi: 10.1371/journal.pone.0251741 (PMC8139490; doi:10.1371/journal.pone.0251741)

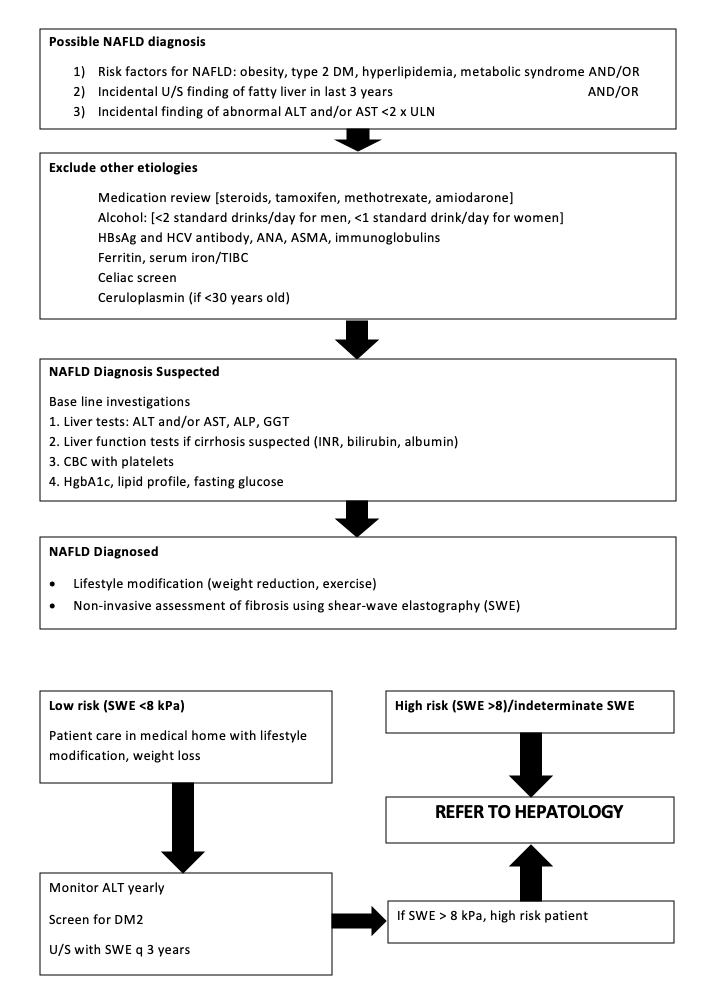

Supplement: S1 Fig — Schematic of the Calgary NAFLD care pathway. (TIF) [file pone.0251741.s005.tif]

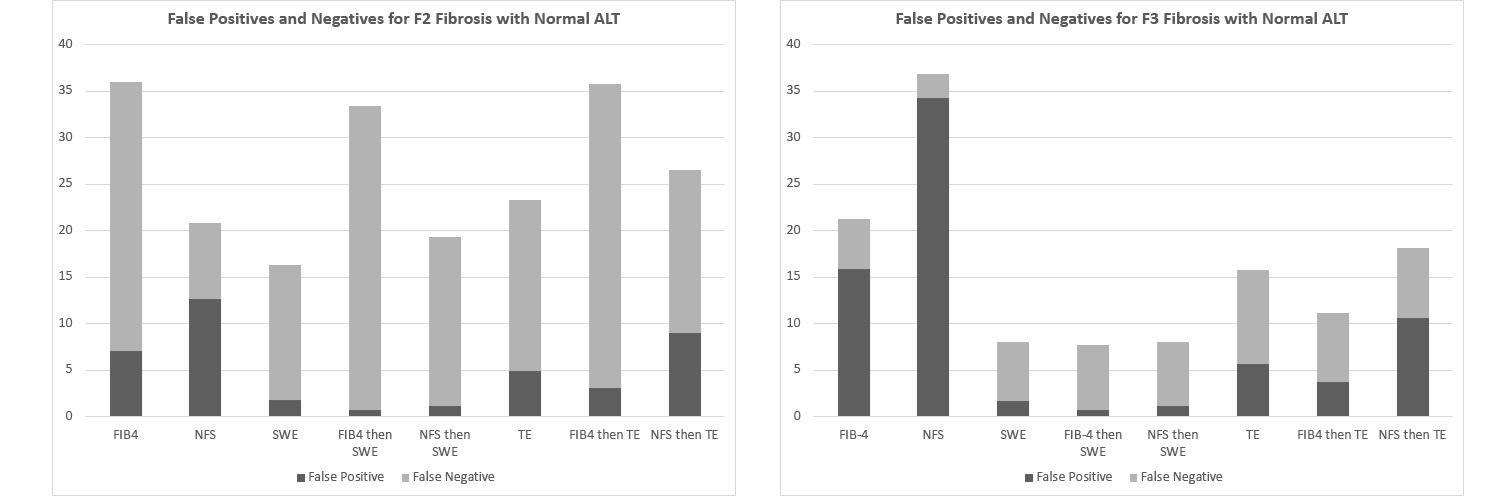

Supplement: S2 Fig — Stratification of incorrect diagnoses based on risk strategies. False positive is considered as being an unnecessary referral for a patient without significant/advanced fibrosis while a false negative was a patient with significant/advanced fibrosis that was not referred to a hepatologist. (TIF) [file pone.0251741.s006.tif]

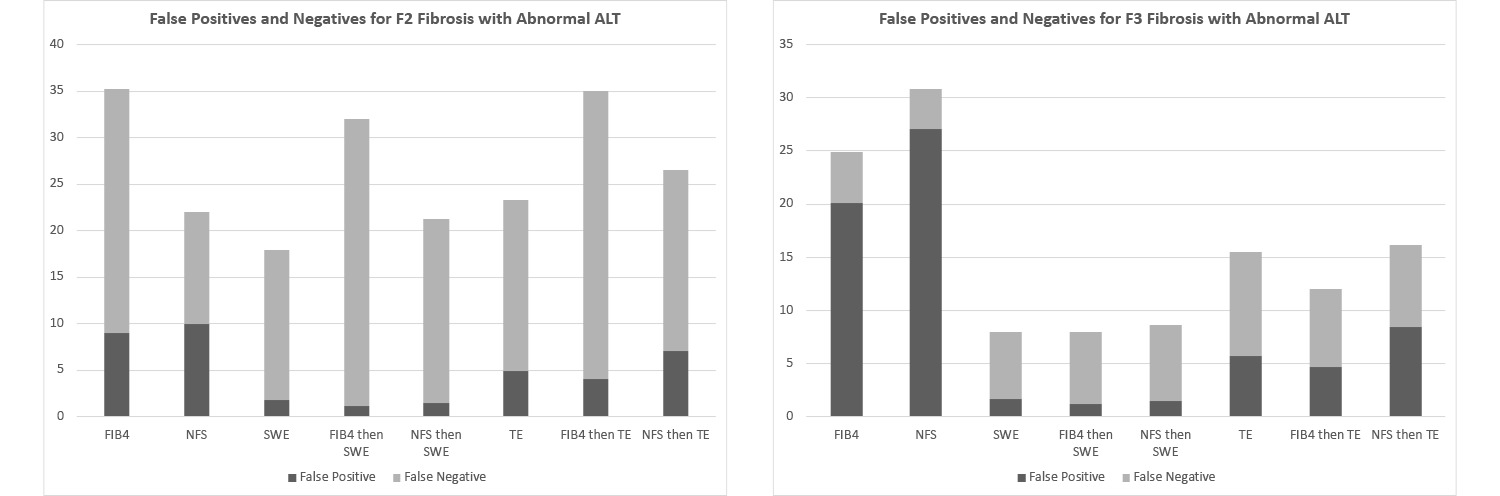

Supplement: S3 Fig — Stratification of incorrect diagnoses based on risk strategies. False positive is considered as being an unnecessary referral for a patient without significant/advanced fibrosis while a false negative was a patient with significant/advanced fibrosis that was not referred to a hepatologist. (TIF) [file pone.0251741.s007.tif]
